# Establishment of a CD46 and desmoglein-2 expressing mouse model for human adenovirus type 55 vaccine evaluation

**DOI:** 10.71150/jm.2605005

**Published:** 2026-09-18

**Authors:** Jung-ah Choi, Eunji Yang, Shing Young Noh, Dae-Im Jung, Yun Jeong Park, Ji Heun Jeong, Hye Yun Jeong, Manki Song, Soon-Hwan Kwon, Sang Hwan Seo

**Affiliations:** 1 Science Unit, International Vaccine Institute, Seoul 08826, Republic of Korea; 2 Department of Infectious Diseases, Armed Forces Medical Research Institute, Daejeon 34059, Republic of Korea

**Keywords:** Human adenovirus type 55, CD46, desmoglein-2, transgenic mice

## Abstract

Human adenovirus type 55 (HAdV-55) is an emerging respiratory pathogen associated with severe pneumonia, for which no licensed vaccines are currently available. The lack of physiologically relevant small-animal models has limited preclinical evaluation of vaccine candidates. In this study, we generated a transgenic mouse model co-expressing human CD46 and desmoglein-2 (DSG-2), key entry receptors for HAdV-55, and evaluated its utility for vaccine efficacy testing. A bicistronic expression system enabling simultaneous expression of CD46 and DSG-2 was constructed and functionally validated in vitro, demonstrating enhanced susceptibility to HAdV-55 infection. The transgenic mice exhibited dose-dependent weight loss, robust viral replication in lung tissues, and characteristic histopathological changes following intranasal challenge, recapitulating key features of human adenoviral pneumonia. Using this model, we assessed the immunogenicity and protective efficacy of an inactivated HAdV-55 (iHAdV-55) vaccine formulated with alum. Vaccination induced strong HAdV-55-specific IgG and neutralizing antibody responses, which increased over time following prime–boost immunization. Upon viral challenge, vaccinated mice showed significantly reduced weight loss and accelerated recovery compared to controls. Viral load analysis demonstrated effective control of viral replication and clearance in vaccinated animals. Collectively, these findings establish the CD46/DSG-2 transgenic mouse as a physiologically relevant and translationally valuable model for HAdV-55 infection and demonstrate that iHAdV-55 vaccination confers robust humoral immunity and protective efficacy. This platform provides a critical tool for the development and preclinical evaluation of adenovirus-targeted vaccines and therapeutics.

## Introduction

Human adenoviruses (HAdVs) are non-enveloped, double-stranded DNA viruses belonging to the family Adenoviridae, responsible for a broad spectrum of clinical manifestations including respiratory, ocular, and gastrointestinal diseases (Lion, 2014). Human adenovirus type 55 (HAdV-55), a recombinant species B virus derived from HAdV-11 and HAdV-14, has emerged as a significant respiratory pathogen associated with severe acute respiratory disease (ARD), particularly among military recruits and immunocompetent adults (Heo et al., 2018; Walsh et al., 2010). This chimeric virus combines the hexon protein from HAdV-11 with the fiber protein from HAdV-14, resulting in enhanced virulence and transmission characteristics (Zhang et al., 2024). Multiple outbreaks have been documented across China and South Korea since 2006, characterized by high morbidity rates and occasional mortality, highlighting an urgent need for effective preventive measures (Ko et al., 2021; Lu et al., 2020; Xie et al., 2025; Yoo et al., 2017; Zhang et al., 2022).

Despite its clinical importance and outbreak potential, no licensed vaccine specifically targeting HAdV-55 is currently available. Although live oral vaccines against HAdV-4 and HAdV-7 have successfully reduced ARD incidence in military populations, their deployment remains geographically limited to the United States military, and cross-protection against HAdV-55 is inadequate due to distinct antigenic properties (Kuschner et al., 2013; Liu et al., 2018; Paris et al., 2014; Tian et al., 2018). Rigorous preclinical evaluation of HAdV-55 vaccine candidates has, however, been fundamentally constrained by the absence of a physiologically relevant small-animal model that faithfully recapitulates human HAdV-55 pathogenesis.

This limitation has been partially addressed by the recent establishment of a cynomolgus macaque model, which offers improved physiological relevance and susceptibility to HAdV-55 infection (Seo et al., 2026). However, non-human primate models are impractical for routine preclinical studies because of their high cost, logistical complexity, and ethical considerations. Therefore, development of a practical and physiologically relevant small-animal model remains a critical unmet need for studies of HAdV-55 pathogenesis and evaluation of medical countermeasures.

The species specificity of human adenoviruses is largely attributable to their dependence on specific cellular receptors for viral attachment and entry. HAdV-55 utilizes desmoglein-2 (DSG2) as a primary attachment receptor and CD46 as an additional entry receptor, whereas murine orthologs do not efficiently support viral infection (Feng et al., 2020). Receptor-humanized mouse models have therefore emerged as a promising strategy to overcome this species barrier. Previous studies using single-receptor transgenic mice, receptor knock-in mice, and multi-receptor humanized models have demonstrated the importance of receptor expression for HAdV-55 susceptibility (Feng et al., 2020; Liu et al., 2024; Wang et al., 2023; Zhou et al., 2024). Building on these findings, we generated a CD46/DSG2 double-transgenic mouse model to provide a practical platform for HAdV-55 infection studies and preclinical evaluation of medical countermeasures.

In this study, we established and characterized a CD46/DSG2 double-transgenic mouse model and evaluated its susceptibility to HAdV-55 infection. Our findings demonstrate that this receptor-humanized model supports HAdV-55-associated respiratory disease and provides a practical platform for studies of viral pathogenesis and preclinical assessment of vaccines and therapeutics.

## Materials and Methods

### Cells

The Vero (African green monkey kidney epithelial) cells (CLB-VERO01WCB-1201, passage 139) used in this study were obtained from the Ministry of Food and Drug Safety (MFDS, Korea), and A549 (Human alveolar basal epithelial) cells (KCLB 10185, passage 40) were obtained from the Korean Cell Line Bank (KCLB, Korea). All cells were cultured in minimum essential medium (MEM; Gibco, USA) supplemented with 10% heat-inactivated fetal bovine serum (FBS; Gibco) and 1% penicillin-streptomycin (Gibco) at 37°C in a humidified 5% CO_2_ incubator. Subculturing was performed every 3 to 4 days.

### Viruses

The human adenovirus type 55 (HAdV-55, AFMRI 41014 strain) used in this study was obtained from the Armed Forces Medical Research Institute (AFMRI, Korea). For virus propagation, A549 cells were seeded into T-175 flasks (Thermo Fisher Scientific, USA) at a density of 1 × 10^7^ cells/flask. After confirming the formation of a monolayer (70–80% confluency) following 24 h of incubation at 37°C and 5% CO_2_, the cells were washed once with serum-free medium and immediately inoculated with the virus. Infection was carried out at a multiplicity of infection (MOI) of 2.5 in serum-free Opti-MEM (Gibco) containing 1% penicillin-streptomycin. When cytopathic effect (CPE) was observed in more than 70% of the cells via microscopy, the culture supernatant was harvested. Viruses were released through two freeze-thaw cycles at -80°C. Subsequently, cells were removed by centrifugation, and the supernatant was filtered using a 0.45 um filter (Corning, USA). Virus purification was performed using a Capto^TM^ Core 700 resin column (Cytiva, USA). After equilibrating the column with phosphate-buffered saline (PBS, Gibco), fractions were collected based on the rise of the ultraviolet (UV) absorbance peak at 280 nm. The collected fractions were concentrated using Amicon^®^ Ultra-15 centrifugal filters (Merck Millipore, USA) and finally stored at -80°C.

The viral titer was measured by plaque assay. A549 cells were seeded in 12-well plates (NUNC, Denmark) at a density of 2.5 × 10^5^ cells/well and allowed to attach for 24 h. After serial 10-fold dilutions of the virus samples, each dilution was inoculated onto the cell monolayer and adsorbed for 2 h at 37°C. Following adsorption, the inoculum was removed, and the cells were overlaid with MEM containing 2% FBS and 0.8% agarose. After solidifying at room temperature, the plates were incubated in a 37°C incubator for 7 days. After incubation, the cells were fixed with 4% paraformaldehyde (Hanlab, Korea), the agarose was removed, and the plaques were visualized by staining with 2.3% crystal violet (Sigma-Aldrich, USA). Viral titers were calculated in plaque-forming units (PFU)/ml according to the following formula:


$$\mathrm{PFU}/\mathrm{ml}=\frac{\mathrm{number}\;\mathrm{of}\;\mathrm{plaques}/\mathrm{well}\times \mathrm{dilution}\;\mathrm{factor}}{\mathrm{inoculum}\;\mathrm{volume}\;(\mathrm{ml})}$$

### Generation and genotyping of double‐receptor mice

A transgenic mouse model was constructed using embryo targeting to insert the human adenovirus receptor genes into the mouse genome while maintaining the cytomegalovirus (CMV) promoters. Human desmoglein2(hDSG2)(NM_001943) and hCD46(NM_002389) gene were constructed, adding the porcine teschovirus-1 2A viral gene (P2A) sequence between hDSG2 and hCD46 cDNA because the P2A gene has a self-cleaving function, it can express hDSG2 and hCD46 protein, respectively. Bovine growth hormone poly A signal sequences were used to terminate mRNA transcription after the coding sequences. After the plasmids were constructed and sequenced without error, mouse embryos were targeted using random recombination into the mouse genome. The random recombination system was transduced into mouse embryo through the micro needle method. The offspring mice were then genotyped by polymerase chain reaction (PCR) of genomic DNA extracted from tail tip samples. The primers used are listed in Table 1 and the corresponding detection primers and band sizes for each mouse genotype are shown in Fig. 1B. After confirming the positive offspring of the double receptors, wild‐type mice were bred to obtain F1 generation mice. Experiments were performed in accordance with the guidelines for Laboratory Animal Care and were approved by the Animal Ethics Committee of armed forces medical research institute (number: AFMRI-202410-A-04).

### In vitro expression of the transgenic construct by MN-CPE

To confirm the expression of the transgenic construct, MLE-12 cells (ATCC cat no. CRL-2110, USA) a murine lung epithelial cell line, were transfected with a transgenic plasmid (control vector, hDSG2, hCD46, and hDSG2-hCD46(double receptor)). MLE-12 cells were cultured at a density of 1.5 × 10^4^ cells/well in a 96-well plate, and on the following day, transfected the transgenic plasmid using lipofectamine LTX at a dose of 200 ng/well plasmid as per manufacturer’s instructions. After 24 h, the cells were infected with 250 PFU/well of adenovirus type 55 and cultured for 5 days. On day 5, the culture medium was removed, and the cells were fixed with 4% paraformaldehyde for 2 h at room temperature (20–24°C), stained with 0.1% crystal violet, washed with distilled water, and air-dried. The plate’s optical density was measured at 590 nm (OD_590_) using a microplate reader (TECAN, Switzerland) to assess the residual cell monolayer. The inhibition rate (%) was calculated as:


$$\mathrm{Inhibition}\;\mathrm{rate}\;(\%)\;=\frac{OD_{sample}-OD_{\mathrm{virus}\;\mathrm{control}}}{OD_{\mathrm{cell}\;\mathrm{control}}-OD_{\mathrm{virus}\;\mathrm{control}}}\times 100$$

where OD_sample_ represents wells containing virus–serum mixtures, OD_virus control_ represents virus-only wells, and OD_cell control_ represents uninfected cell-only wells.

### Histological analysis of transgene expressions in lung

Lung tissues collected after infection were fixed in 10% neutral-buffered formalin (NBF). The fixed tissues were processed using a standard histological protocol, embedded in paraffin, and sectioned to prepare tissue slides. For staining, the sections were incubated overnight at 4°C with a human DSG-2 (hDSG-2) primary antibody (Cat. No. ab150372, Abcam). After three washes with PBS, the sections were incubated for 1 h with a peroxidase-conjugated rabbit IgG secondary antibody (Cat. No. PK-4001, VECTASTAIN). The slides were visualized using a DAB Substrate Kit (Cat. No. SK-4100, Vector Laboratories). The intensity of antibody staining was quantified using ImageJ software (NIH, USA).

### Preparation of HAdV-55 vaccine candidate

HAdV-55 virus for vaccine production was cultured using Vero cells and Opti-MEM. Harvesting and purification of the virus were performed as described above via freeze-thaw cycles, centrifugation, and filtration using a 0.2 um Sartopore^®^ 2 filter (Sartorius, Germany). Subsequently, purification was carried out using a Capto^TM^ Core 700 resin column; after equilibration with PBS, fractions were collected based on the 280 nm UV absorbance peak. For virus inactivation, formaldehyde (final 0.1M) was added to the purified virus, followed by continuous stirring at 37°C for 24 h. Residual formaldehyde was removed using an ultrafiltration/diafiltration (UF/DF, Sartorius) system by buffer exchange with 15 volumes of PBS. The final vaccine formulation was adjusted to an antigen concentration of 1,000 internal unit (IU)/ml, and aluminum hydroxide gel (Sigma-Aldrich) was added as an adjuvant to a final concentration of 0.5 mg/ml (5% v/v), after which the final volume was adjusted using PBS. The final mixture was stirred at 120 rpm for 60 min and then aseptically filled into vials at 1.2 ml/vial and stored at 2–8°C. All vaccine manufacturing and vialing procedures were performed at the K-Bio CMO Center of the Korea Vaccine Global Industrialization Foundation Project Group (Korea).

### Mouse infection study

Twelve- to sixteen-week-old hCD46/hDSG2 double transgenic (TG) mice were used for the virus challenge dose study, with eight mice allocated per experimental group. The animals were sourced from the AFMRI and maintained in the animal facility at the International Vaccine Institute (IVI). All animal procedures, including breeding and experimental protocols, were reviewed and approved by the Institutional Animal Care and Use Committee (IACUC) of the IVI (IACUC PN 2023-006 and IACUC PN 2023-007, respectively).

To evaluate the pathogenicity of HAdV-55 according to the infectious dose, mice were anesthetized via an intraperitoneal (i.p.) injection of a ketamine (100 mg/kg; Yuhan, Korea) and xylazine (10 mg/kg; Bayer Korea, Korea) mixture. The mice were then challenged intranasally (i.n.) with 1.5 × 10^9^ PFU, 1.5 × 10^8^ PFU, or 1.5 × 10^7^ PFU of HAdV-55, prepared from a stock concentration of 3 × 10^10^ PFU/ml, in a total volume of 50 μl. Following the challenge, body weight was monitored daily for 10 days. On days 2 and 4 post-infection (dpi), two mice/group were euthanized by an overdose of the ketamine/xylazine mixture to harvest lung samples. The remaining four mice/group were euthanized on 10 dpi for lung sample collection. All harvested lungs were utilized for the determination of viral titers and histopathological analysis.

For inactivated virus inoculation study, pure adenovirus samples underwent chemical inactivation using 0.01 M formaldehyde (Sigma-Aldrich, USA) at 37°C during 16 h, while five diafiltration rounds against PBS inside an Amicon Ultra Centrifugal Filter Unit (10 kDa MWCO; Merck Millipore, USA) removed residual formaldehyde levels. Remaining formaldehyde quantities checked against an acetylacetone-based colorimetric assay plus a formaldehyde reference standard (AccuStandard, USA). Total viral inactivation met criteria via plaque assay, plus antigen stability underwent checks using a commercial adenovirus Hexon enzyme-linked immunosorbent assay (ELISA) kit (Creative Diagnostics, USA) per specific manufacturer instructions. Comparative antigen potency reached values using the identical pre-inactivation virus batch provided as the primary reference standard. During vivo evaluation, hCD46/hDSG2 double-transgenic mice received ketamine (100 mg/kg) and xylazine (10 mg/kg) plus intranasal doses containing either live or formalin-inactivated HAdV-55 (1.5 × 10^9^ PFU/mouse). Two mice/group faced euthanasia on days 2 and 4 post-inoculation, plus lung tissues underwent collection for histopathological studies following fixation in 10% neutral-buffered formalin.

### Quantitative polymerase chain reaction for lung virus titration

The amount of virus in lung tissue samples was analyzed by quantitative reverse transcription real-time PCR (qRT-PCR) after extracting DNA using the Viral Gene-spin^TM^ kit (iNtRON, Korea). For analysis, TB Green Advantage qPCR Premix (Takara Bio, Japan) and a specific primer set (Forward: 5'-CAGTGGATTGCAGAAGGT-3', Reverse: 5'-AGCATAAATCGGTTTACTTTCTTC-3') were used. The amplification reaction was performed using the QuantStudio^TM^ 7 Flex (Thermo Fisher Scientific) with initial denaturation at 95°C for 30 s, followed by 40 cycles of 95°C for 3 s and 57°C for 31 s. Following amplification, melting curve analysis was performed, and the viral viral DNA copy number (GE, genomic equivalent) was quantified based on a standard curve generated with a standard template of known concentration.

### Plaque assay

A plaque assay was performed to measure the infectious viral titer in lung tissue. Each collected lung tissue sample was serially 2-fold diluted using serum-free medium. The prepared dilutions were inoculated onto A549 cell monolayers in 12-well plates and infected for 2 h at 37°C. After removing the inoculum, 1 ml of MEM containing 2% FBS and 0.8% agarose was overlaid. After solidifying at room temperature, the plates were incubated at 37°C and 5% CO_2_ for 7 days. Following incubation, cells were fixed with 4% paraformaldehyde, the agarose was removed, and staining was performed with 2.3% crystal violet. Plaques were manually counted to calculate the viral titer.

To analyze HAdV-55-specific neutralizing antibody (nAb) titers, a plaque reduction neutralization test (PRNT) was performed. Serum samples were serially 2-fold diluted starting from an initial 1:20 dilution in serum-free MEM. Each serum dilution was mixed with HAdV-55 virus suspension at a 1:1 (v/v) ratio and reacted at 37°C for 60 min to induce neutralization. The neutralized serum-virus mixture was then added to the A549 cell monolayers in 12-well plates and infected for 1 h at 37°C, after which the inoculum was removed. Subsequently, 1 ml of MEM containing 2% FBS and 0.8% agarose was overlaid and allowed to solidify, followed by incubation at 37°C and 5% CO_2_ for 7 days. After incubation, the cells were fixed with 4% paraformaldehyde and stained with 2.3% crystal violet to count the plaques. The neutralizing antibody titer (PRNT_50_) was defined as the reciprocal of the highest serum dilution that reduced the number of plaques by at least 50% compared to the virus-only control.

### Histological analysis

Lung tissues harvested post-infection were fixed in 10% neutral buffered formalin. The fixed tissues underwent a standardized tissue processing sequence, were embedded in paraffin blocks, and subsequently sectioned (cutting) to prepare tissue slides. The sections were then stained with Hematoxylin and Eosin (H&E) for morphological evaluation.

To ensure objectivity, a blinded assessment was conducted by an experienced pathologist. The severity of lung injury was evaluated based on two primary histopathological parameters: edema and inflammatory cell infiltration. All qualitative and quantitative findings observed in the H&E-stained slides were systematically recorded according to the following grading scale: no remarkable (-), minimal (±), slight (+), and moderate (++). All histopathological procedures and analyses were conducted at the contract research organization Biotoxtech (Korea).

### Enzyme-linked immunosorbent assay

To analyze HAdV-55-specific binding antibodies, 96-well Maxisorp plates (Nunc, Denmark) were coated with purified HAdV-55. The virus, with an initial concentration of 1 × 10^10^ pfu/ml, was diluted to a working concentration of 2.5 × 10^7^ pfu/ml in PBS (Gibco) and added to the plates at 100 μl/well. After overnight incubation at 4°C, the plates were washed three times with wash buffer (PBS containing 0.05% Tween 20 [Sigma-Aldrich]). Blocking was performed by adding 100 μl of blocking buffer (2% bovine serum albumin (BSA) [Sigma-Aldrich] in PBS containing 0.05% Tween 20) to each well, followed by incubation for 1 h at 37°C. After blocking, the wells were washed three times with wash buffer. Mouse sera samples, initially diluted 1:100 in antibody buffer (0.5% BSA and 0.05% Tween 20 in PBS), were added to the wells at 100 μl/well, followed by 5-fold serial dilutions. The plates were incubated overnight at 4°C. Subsequently, the wells were washed three times with wash buffer, and 100 μl of goat anti-mouse horseradish peroxidase-labeled IgG (Southern Biotech, USA), diluted 1:3,000 in antibody buffer, was added to each well and incubate for 1 h at 37°C. After the final incubation, the wells were washed three times with wash buffer. For color development, 100 μl of 3,3',5,5'-tetramethylbenzidine substrate (Millipore, Germany) was added to each well. The reaction was terminated by adding 50 μl of 0.5 N HCl (Merck, Germany) per well. The absorbance (OD, optical density) was measured at 450 nm using a SpectraMax 340PC384 microplate reader (Molecular Devices, USA). The resulting data were analyzed with SoftMax Pro 7.1 software (Molecular Devices) to calculate the log_2_ antibody titers.

### Protection study

Six- to eight-week-old hCD46/hDSG2 double TG mice were used for the protection study, with fourteen mice allocated per experimental group. The mice were sourced and maintained under the same conditions as described in the challenge dose study.

To evaluate the immunogenicity and protective efficacy of the inactivated HAdV-55 vaccine, mice were immunized intramuscularly (i.m.) twice at a 4-weeks interval. Blood samples were collected three times at 2-week intervals starting after the prime immunization to evaluate antigen-specific antibody responses and neutralizing activity via ELISA and PRNT, respectively. Four weeks after the final immunization, mice were anesthetized and challenged i.n. with 1.5 × 10^9^ PFU of HAdV-55 in a total volume of 50 μl, following the same procedure and anesthetic regimen as described above. Following the challenge, body weight and survival rates were monitored daily for 14 days. On 2, 4, and 7 dpi, three mice/group were euthanized by an overdose of the ketamine/xylazine mixture to harvest lung samples for viral titer determination and histopathological analysis.

### Statistical analysis

All data are presented as Means ± standard deviation (SD). Group comparisons were performed using the unpaired Student's t-test. For comparison of body weight changes, group differences over time were assessed by repeated-measures two-way ANOVA with group as the between-subject factor and day post-infection as the within-subject factor, implemented using a linear mixed-effects model with REML estimation to accommodate the unbalanced longitudinal design arising from early humane-endpoint removal. Effect sizes are reported as partial eta-squared (η²p). Post-hoc pairwise comparisons between groups were performed using independent-samples t-tests with Bonferroni correction applied across all pairwise contrasts within each figure. All analyses were performed in Python (v3.12) using the pingouin (v0.6.1) and SciPy (v1.x) libraries. Statistical significance was defined as *P* < 0.05.

## Results

### Construction and validation of CD46/Desmoglein-2 expression system and generation of transgenic mice

To construct the transgenic expression system, hDSG2 and hCD46 cDNAs were cloned into the pcDNA3.1 vector, with a P2A sequence inserted between them to enable independent translation of both hDSG2 and hCD46 proteins from a single transcript (Fig. 1A).

To validate expression and function of the transgenic construct, MLE-12 cells were transfected with control vector, hDSG2 alone, hCD46 alone, or hDSG2-hCD46, then challenged with adenovirus type 55 (HAdV-55) at multiplicities of infection (MOI) of 100, 50, 25, and 12.5. Following a five-day incubation period, cytopathic effects (CPE) were quantified by morphological analysis. Cells co-expressing hDSG2 and hCD46 exhibited significantly enhanced infection rates compared to single-receptor expressing cells across all viral concentrations tested (*P* < 0.05) (Fig. 1B), demonstrating that HAdV-55 infectivity is substantially improved by dual receptor co-expression.

To generate transgenic animals, the construct was introduced into fertilized mouse eggs via microinjection, resulting in 27 live births. Molecular genotyping identified two transgenic founders (lineage 1 and lineage 8). Lineage 1 successfully produced F1 offspring through wild-type backcrossing and was selected for further study. Lineage 8 was discontinued due to breeding failure (no pregnancy achieved) (Fig. 1C). All subsequent experiments were performed using lineage 1 transgenic mice. To confirm transgene expression in the lungs of the transgenic mice, hDSG2 expression in pulmonary epithelial cells was examined by immunohistochemistry. hDSG2 staining was detected in the pulmonary epithelial cells of dTG mice (Fig. 1D). Quantitative analysis of staining intensity using ImageJ further demonstrated significantly higher hDSG2 expression in dTG mice compared with C57BL/6 mice (*P* < 0.01), confirming the expression of the hDSG2 transgene in the lungs of dTG mice.

### Characterization of a CD46/Desmoglein-2 transgenic mouse model for HAdV-55 infection

Following generation of CD46/DSG-2 double transgenic mice, animals were characterized for their susceptibility to HAdV-55 infection. To first establish whether transgenic receptor expression confers susceptibility to HAdV-55, CD46/DSG-2 TG mice and wild-type C57BL/6 controls were intranasally inoculated with HAdV-55 at 1.5 × 10^9^ PFU and monitored for body weight change over seven days (Fig. 2A). TG mice exhibited significantly greater body weight loss and impaired recovery compared with wild-type (WT) controls (*P* < 0.05). These findings confirm that human CD46 and DSG-2 co-expression renders mice permissive to HAdV-55-induced morbidity in a manner not reproducible in WT C57BL/6 mice.

To further characterize the model, transgenic mice were intranasally inoculated with serial dilutions of HAdV-55 (1.5 × 10^7^, 1.5 × 10^8^, and 1.5 × 10^9^ PFU), and body weight changes, lung viral titers, and viral genome copies were monitored according to the experimental scheme in Fig. 2B. HAdV-55 infection resulted in dose-dependent body weight loss (*P* < 0.001, Fig. 2C). Mice receiving the highest viral dose (1.5 × 10^9^ PFU) demonstrated the most pronounced acute weight reduction, declining to approximately 85% of baseline within 2–3 dpi before initiating partial recovery. Intermediate-dose recipients (1.5 × 10^8^ PFU) exhibited moderate weight loss with attenuated kinetics, while low-dose recipients (1.5 × 10^7^ PFU) showed minimal clinical signs. Mock-infected control animals maintained stable body weights throughout the observation period, confirming that weight loss was virus-specific rather than procedure-related.

Plaque assays on lung homogenates revealed dose-dependent viral loads peaking at 2 dpi, with the highest-dose group yielding titers of approximately 10⁶ PFU/ml (Fig. 2D). Viral titers declined substantially by 4 dpi across all dose groups and approached the limit of detection by 10 dpi. qRT-PCR analysis corroborated these findings, with viral genome copy numbers highest in the 1.5 × 10^9^ PFU group at 2 dpi and reduced to baseline or undetectable levels by 10 dpi across all experimental groups (Fig. 2E). The concordance between plaque assay and RT-PCR data confirms dose-dependent pulmonary viral retention followed by progressive clearance.

Collectively, these data demonstrate that CD46/DSG-2 transgenic mice recapitulate key features of HAdV-55 infection, including receptor-dependent susceptibility, dose-dependent clinical morbidity, and dose-dependent pulmonary viral retention with subsequent clearance. This model provides a validated platform for preclinical evaluation of HAdV-55 vaccine candidates and antiviral interventions.

### Histopathological analysis of HAdV-55-induced lung pathology

To evaluate the pathological consequences of HAdV-55 infection, lung tissues were collected at 2, 4, and 10 dpi from transgenic mice infected with different viral doses and examined by hematoxylin and eosin (H&E) staining (Fig. 3). Histopathological changes were scored for inflammation and edema on a scale of 0–4 to quantify disease severity. HAdV-55 infection induced dose-dependent pulmonary pathology with distinct temporal patterns. At 2 dpi, mice infected with the highest dose (1.5 × 10^9^ PFU) exhibited severe inflammatory infiltrates characterized by dense accumulation of inflammatory cells in peribronchiolar and perivascular regions (yellow arrows), accompanied by prominent alveolar wall thickening and edema formation. The inflammation score reached 3.0 ± 0.2, with concurrent edema scoring 2.8 ± 0.3. Intermediate-dose infected animals (1.5 × 10^8^ PFU) showed moderate inflammatory changes with scores of 2.0 ± 0.1 for inflammation and 2.2 ± 0.2 for edema, while low-dose recipients (1.5 × 10^7^ PFU) demonstrated mild pathological alterations. By 4 dpi, histopathological severity remained elevated in the high-dose group, maintaining inflammation and edema scores of approximately 3.0 and 2.0, respectively. However, intermediate and low-dose groups showed progressive resolution of inflammatory changes, with reduced cellular infiltration and improved alveolar architecture. Areas of focal pneumonia and bronchiolar epithelial damage were still evident in severely affected animals (black arrowheads). At 10 dpi, significant resolution of pathological changes was observed across all infection groups. The high-dose group showed marked improvement with inflammation scores declining to 1.0 ± 0.3 and edema scores to 0.5 ± 0.2. Lung architecture appeared largely restored, with minimal residual inflammatory infiltrates and normalized alveolar wall thickness. Low and intermediate-dose groups demonstrated near-complete histological recovery, with scores approaching those of uninfected controls. Mock-infected control animals maintained normal lung histology throughout the observation period, with preserved alveolar architecture, minimal inflammatory cell presence, and inflammation/edema scores consistently at or near zero.

To determine whether the observed effects were attributable to active viral infection or simply to intranasal exposure to a large number of viral particles, human CD46/DSG-2 transgenic mice were intranasally inoculated with PBS, live HAdV-55, or inactivated HAdV-55 and examined at 2 and 4 dpi (Fig. S1). Inactivated HAdV-55 induced mild pulmonary inflammation and edema, whereas live HAdV-55 caused markedly stronger inflammatory cell infiltration, edema, and alveolar septal thickening. Histological scores for inflammation and edema were consistently higher in the live HAdV-55 group than in the PBS or inactivated virus groups, indicating that the pulmonary lesions were primarily attributable to active viral infection.

Collectively, these findings demonstrate that HAdV-55 infection in CD46/DSG-2 transgenic mice induces dose-dependent acute pulmonary pathology that resolves over time, recapitulating key features of human adenoviral pneumonia and validating the utility of this model for vaccine efficacy studies.

### Evaluation of the protective efficacy of inactivated HAdV-55 vaccine candidate using human CD46 and DSG-2 transgenic mice

To assess the protective efficacy of the inactivated HAdV-55 (iHAdV-55) vaccine, CD46/DSG-2 TG mice were immunized with iHAdV-55 formulated with alum (AH) following a prime–boost regimen (weeks 0 and 4) (Fig. 4A). Serum samples were collected at weeks 2, 4, and 6 post-immunizations to evaluate humoral immune responses, and mice were subsequently challenged with HAdV-55 at week 8. Immunization with iHAdV-55 + AH elicited robust antigen-specific antibody (Ab) responses. HAdV-55-specific IgG titers were readily detectable at 2 weeks post-immunization and increased progressively through weeks 4 and 6 (Fig. 4B). In contrast, PBS-treated control mice exhibited no detectable IgG responses. The magnitude of the IgG response in the vaccinated group reached high titers by week 6, indicating effective priming and boosting of the humoral immune response. Consistent with binding Ab (bAb) responses, neutralizing Ab (nAb) titers were strongly induced in vaccinated animals (Fig. 4C). PRNT_50_ values increased over time, reflecting the maturation of functional Ab responses capable of inhibiting viral infection. No HAdV-55-specific nAb response was detected in PBS-treated mice at any time point. These results demonstrate that iHAdV-55 vaccination induces potent humoral immunity, including both bAb and nAb.

To evaluate protective efficacy, vaccinated and control mice were challenged intranasally with HAdV-55, and clinical and virological parameters were assessed (Fig. 4A). PBS-immunized mice experienced rapid weight loss, declining to approximately 83% of initial body weight by day 2 post-infection, with gradual recovery over 14 days but not reaching 100% of initial body weight (Fig. 5A). In contrast, iHAdV-55 + AH-immunized mice showed less initial weight loss, declining to only 88% of initial body weight, with faster recovery returning to near baseline by day 13 post-infection. Viral replication in lung tissues was quantified by both plaque assay and qRT-PCR at 2, 4, and 7 dpi (Fig. 5B). Viral replication in lung tissues was quantified by plaque assay and qRT-PCR at 2, 4, and 7 dpi. In plaque assays, vaccinated mice exhibited markedly reduced infectious viral loads at 2 dpi (5.02 Log_10_ PFU/ml vs. 7.32 Log_10_ PFU/ml in PBS controls) and 4 dpi (2.01 Log_10_ PFU/ml vs. 4.79 Log_10_ PFU/ml), representing 2.3 and 2.77 log reductions, respectively. qRT-PCR analysis revealed significantly lower viral genome levels in vaccinated mice throughout infection, with 2.72 Log_10_ GE/μg at 2 dpi (vs. 5.98 in PBS controls) and 1.93 Log_10_ GE/μg at 4 dpi (vs. 5.01 in PBS controls). By 7 dpi, vaccinated mice achieved near-complete viral clearance, while PBS controls maintained approximately 2.22 Log_10_ GE/μg viral genomes. These results demonstrate superior viral control and sustained suppression in vaccinated mice.

Taken together, these findings demonstrate that iHAdV-55 vaccination induces strong humoral immune responses and confers protective efficacy against HAdV-55 challenge in a human receptor-expressing transgenic model. The CD46/DSG-2 transgenic mouse system thus represents a valuable and translationally relevant platform for preclinical evaluation of adenovirus-targeted vaccines and therapeutics.

## Discussion

In this study, we established a human CD46/DSG2 double-transgenic mouse model and demonstrated its utility for evaluating protective efficacy of HAdV-55 vaccine candidate. Our findings show that human CD46/DSG2 double-transgenic mice are susceptible to HAdV-55 infection, resulting in measurable clinical illness, pulmonary viral recovery, and histopathological lung injury following intranasal challenge. Using this model, we further demonstrated that a formalin-inactivated HAdV-55 vaccine formulated with alum induced robust humoral immunity and significantly reduced disease severity and viral burden after challenge. Collectively, these data support the translational value of this model for preclinical vaccine and therapeutic development against HAdV-55 infection.

A major obstacle in HAdV-55 research has been the lack of practical small-animal models that faithfully reproduce host susceptibility. Human adenoviruses are strongly restricted species, largely because efficient infection depends on specific host cell receptors and intracellular compatibility factors. Species B adenoviruses, including HAdV-55, utilize DSG2 as a major attachment receptor and CD46 as an additional entry mediator, whereas murine orthologs do not support infection efficiently (Cupelli et al., 2010; Feng et al., 2020). Previous mechanistic studies showed that expression of human DSG2 and CD46 markedly enhances HAdV-55 entry, with DSG2 playing a dominant role in receptor usage (Feng et al., 2020). Our in vitro data is consistent with these observations, as co-expression of both receptors produced greater susceptibility than single-receptor expression alone. These results suggest that simultaneous receptor expression more accurately reflects the multistep viral attachment and entry process than single-transgene systems.

The present model should be considered in the context of previously reported receptor-expressing mouse systems. Feng et al. (2020) generated separate hDSG2 and hCD46 single-receptor transgenic lines and demonstrated that hDSG2 transgenic mice confer substantially greater in vivo HAdV-55 susceptibility than hCD46 transgenic mice (Feng et al., 2020). Liu et al. (2024) subsequently employed the hDSG2 single-receptor line from Feng et al. (2020) to evaluate neutralizing monoclonal antibodies against HAdV-55 in vivo, further demonstrating the utility of receptor-transgenic systems for therapeutic assessment (Liu et al., 2024). Wang et al. (2023) developed a tri-receptor transgenic model co-expressing hDSG2, hCD46, and coxsackievirus and adenovirus receptor, called CAR, designed for broad-spectrum adenovirus research across multiple serotypes; however, the complexity of tri-receptor expression is less suited to mechanistically focused evaluation of HAdV-55-specific interventions (Wang et al., 2023). More recently, Zhou et al. (2024) reported hDSG2/hCD46 double knock-in (KI) mice generated by targeted genomic insertion and demonstrated that dual-receptor KI mice exhibit greater susceptibility to species B adenovirus infection than single-receptor transgenic mice, providing independent in vivo support for the superiority of dual-receptor co-expression (Zhou et al., 2024). That model was validated primarily for HAdV-7, however, and its direct applicability to HAdV-55 pulmonary pathogenesis and vaccine evaluation has not been characterized. The present model employs a bicistronic CMV-driven transgenic construct that enables simultaneous co-expression of hDSG2 and hCD46 from a single integration site via a P2A self-cleaving peptide, offering a technically accessible and reproducible platform for routine HAdV-55 preclinical screening. We acknowledge that direct in vivo comparison between single- and dual-receptor transgenic lines was not performed in the present study, and that CMV promoter-driven expression may not fully replicate endogenous receptor distribution in the respiratory epithelium. Accordingly, this model is most appropriately utilized as a practical first-line evaluation tool for candidate prioritization, complementary to KI models and nonhuman primate studies rather than a replacement for either.

The in vivo phenotype observed in our model further supports its biological relevance. Intranasal challenge produced dose-dependent weight loss, high early lung viral titers, inflammatory infiltrates, edema, and subsequent recovery. This pattern resembles the acute but self-limited respiratory disease reported in immunocompetent human HAdV infections, while also capturing features associated with severe HAdV-55 pneumonia, including intense pulmonary inflammation. Clinical reports from China and South Korea have described HAdV-55 as a cause of severe community and military outbreaks, occasionally requiring intensive care support (Heo et al., 2018; Ko et al., 2021; Zhang et al., 2022). The ability of our model to reproduce quantifiable respiratory pathology provides an important advantage over conventional mouse strains that are minimally permissive to infection.

Several animal systems have previously been explored for adenovirus pathogenesis, including cotton rats, Syrian hamsters, tree shrews, and nonhuman primates, each with distinct advantages and limitations (Bertzbach et al., 2021). Nonhuman primate models may provide high physiological relevance but are costly and low throughput. Recently, a cynomolgus macaque model of HAdV-55 respiratory disease was reported, supporting its usefulness for translational studies (Seo et al., 2026). However, routine vaccine screening requires scalable and genetically tractable small-animal systems. In this context, our double-transgenic model may complement macaque studies by enabling efficient candidate prioritization before advanced development.

Using this platform, we demonstrated that an inactivated whole-virion HAdV-55 vaccine adjuvanted with alum elicited strong binding and neutralizing antibody responses. Neutralizing antibodies increased substantially after booster immunization, consistent with prior studies showing that prime–boost regimens improve humoral immunity against HAdV-55 (Seo et al., 2024). Although the two-dose immunization regimen with the inactivated HAdV-55 vaccine induced robust neutralizing antibody responses, complete protection against viral challenge was not achieved. One possible explanation is the relatively high challenge dose used in this study, which was selected to establish a consistent and measurable infection in the transgenic mouse model. This challenge dose likely exceeds the level of natural exposure and therefore represents a stringent test of vaccine efficacy. Nevertheless, vaccinated animals showed reduced lung viral loads and attenuated body weight loss compared with PBS-treated controls, indicating that the vaccine conferred partial but meaningful protection. These findings suggest that while neutralizing antibodies contribute substantially to protection, additional immune mechanisms, including cellular immune responses, may also be important for achieving optimal protection against HAdV-55 infection. Future studies evaluating alternative vaccine formulations, adjuvants, immunization schedules, and challenge doses may further improve protective efficacy in this model.

In addition to vaccine evaluation, this model may be valuable for testing antiviral antibodies and other prophylactic approaches. Recent studies have shown that neutralizing monoclonal antibodies can protect against HAdV-55 infection in receptor-transgenic mice and tree shrews, reinforcing the importance of receptor-appropriate small-animal systems for biologic evaluation (Liu et al., 2024). More broadly, the utility of humanized receptor models has been demonstrated in adenovirus research and supports the concept that host entry factors are critical determinants of infection outcome. Our model extends this principle by combining two relevant receptors in a single mouse line, thereby improving biological relevance for species B adenovirus infection.

This study has several important limitations. First, although our murine model supports susceptibility to HAdV-55 infection, recovery of infectious virus from lung tissues, and development of pulmonary disease following viral challenge, significant differences exist between murine and human immunity and physiology. Human adenoviruses are generally considered species-restricted pathogens and do not efficiently complete their replication cycle in murine hosts because of limitations in both receptor usage and intracellular host factors (Blair et al., 1989). Consistent with this characteristic, lung viral titers gradually declined after Day 2 post-infection in our study. Therefore, recovery of infectious viruses from lung tissues should not be interpreted as definitive evidence of productive viral replication. Additionally, transgene expression driven by an artificial promoter may not fully recapitulate native CD46 and DSG-2 receptor distribution in the human airway. Consequently, this model is best utilized as a practical first-line screening platform for rapid and cost-effective evaluation of vaccine and therapeutic candidates, enabling prioritization of promising approaches for validation in nonhuman primates with greater physiological relevance. Second, our evaluation primarily focused on humoral immunity; consequently, mucosal responses, T-cell immunity, and protection durability require further investigation in future studies.

In conclusion, we developed a practical and biologically relevant CD46/DSG2 double-transgenic mouse model that overcomes a key barrier in HAdV-55 research. The model supports reproducible respiratory infection and enables quantitative assessment of vaccine efficacy. Using this platform, we demonstrated that an alum-adjuvanted inactivated HAdV-55 vaccine induces potent neutralizing antibodies and protects against pulmonary challenge. These findings provide an important foundation for accelerated development of vaccines and therapeutics against HAdV-55.

## Figures and Tables

**Fig. 1.**
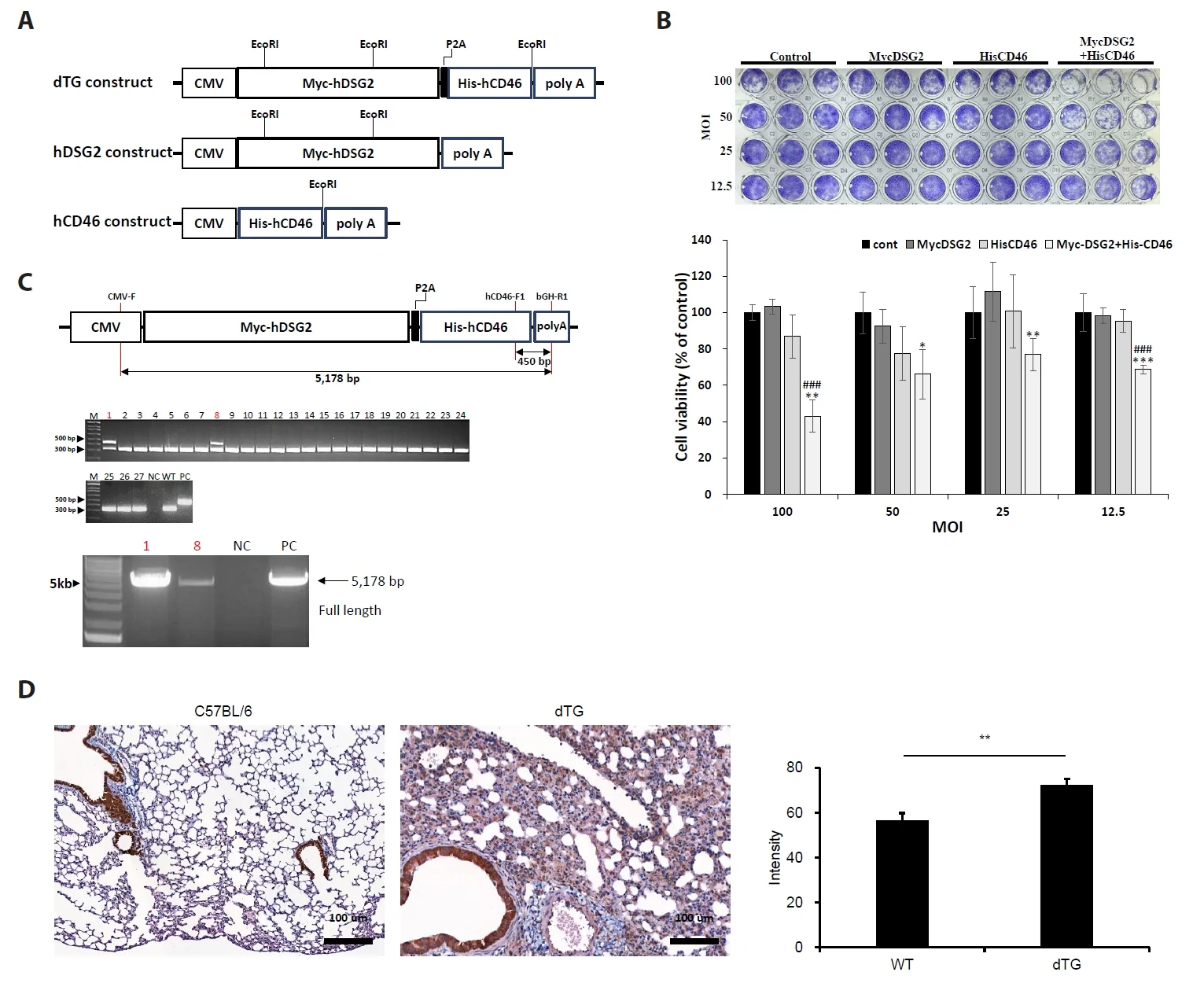
Construction and functional validation of bicistronic CD46/DSG2 expression system. (A) Schematic representation of plasmid constructs. The dual transgene (dTG) construct contains Myc-tagged human desmoglein-2 (hDSG2) and His-tagged human CD46 (hCD46) separated by a P2A self-cleaving peptide under the control of the CMV promoter, followed by a polyadenylation signal (poly A). Restriction enzyme sites (EcoRI) used for cloning are indicated. (B) Functional assessment of receptor-mediated viral susceptibility. Crystal violet staining of infected cell monolayers at different multiplicities of infection (MOI: 100, 50, 25, and 12.5). Quantification of cell viability is presented as percentage relative to control. Data represents SD. Statistical significance is indicated as *P < 0.05, **P < 0.01, ***P < 0.001 versus MycDSG2, and ^###^*P* < 0.001 versus HisCD46. (C) Validation of bicistronic construct and transgene integration. Upper panel shows the full-length dTG construct (~5,178 nucleotides) with primer binding sites. Middle panel: PCR screening of transgenic candidates demonstrating expected amplicons. Lower panel: agarose gel electrophoresis confirming full-length fragment (~5,178 nucleotides) in positive clones (PC), with negative control (NC) and molecular marker (M) indicated. (D) Protein expression of hDSG2 in pulmonary epithelial cells of C57BL6 and dTG by immunohistochemistry. The stained intensity was calculated by Image J software. **P < 0.01.

**Fig. 2.**
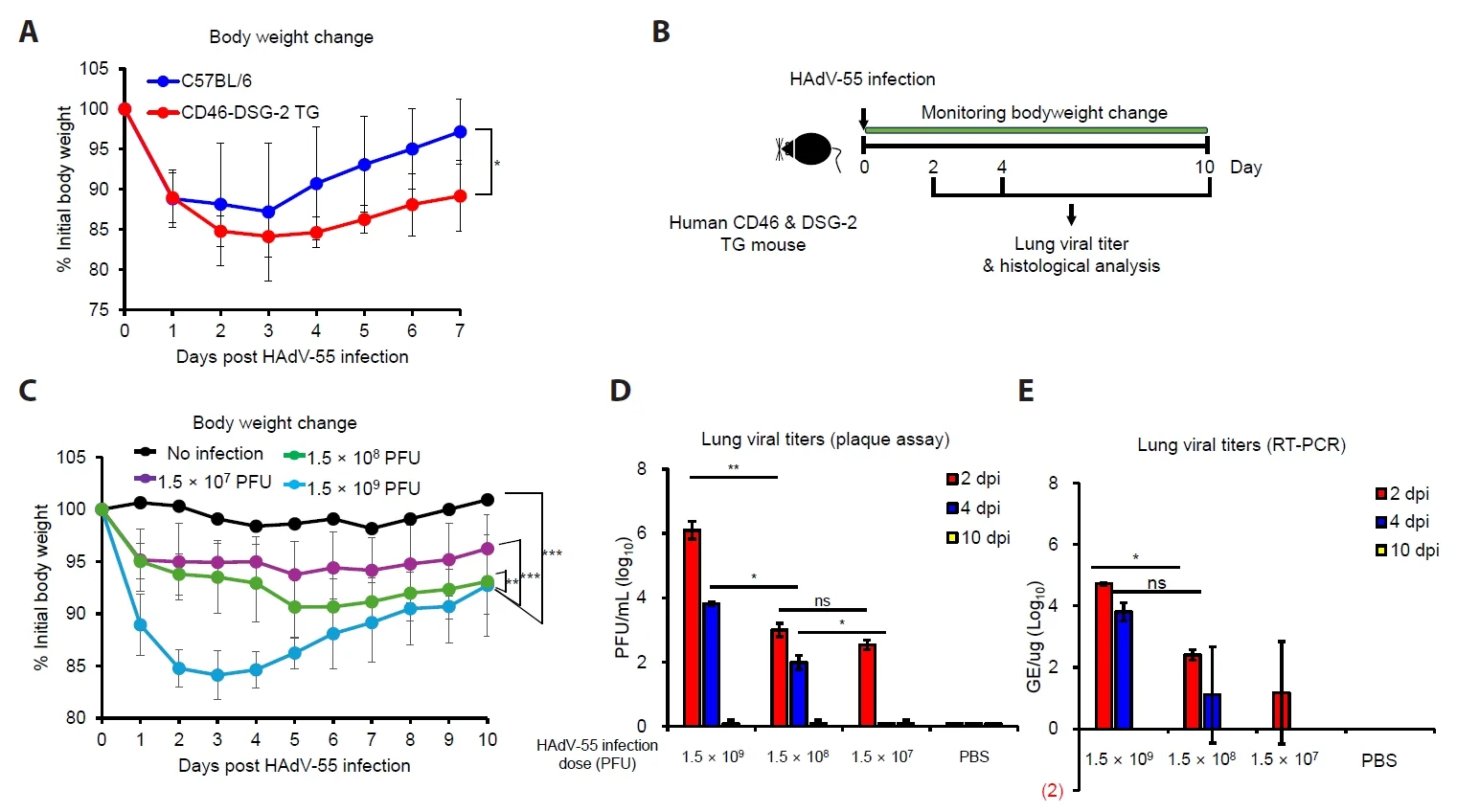
Susceptibility of CD46/DSG-2 double transgenic mice to HAdV-55 infection. (A) Body weight changes in wild-type C57BL/6 and CD46/DSG-2 TG mice following intranasal inoculation with HAdV-55 at 1.5 × 10⁹ PFU (WT, n = 5; TG, n = 8). (B) Schematic of the experimental design for panels C–E. (C) Body weight changes in TG mice inoculated with HAdV-55 at the indicated doses or PBS (n = 8/group). (D, E) Lung viral titers determined by plaque assay (D) and qRT-PCR (E) at 2, 4, and 10 dpi (n = 2–4/time point). Data are presented as Mean ± SD. Statistical comparisons were performed by repeated-measures two-way ANOVA with Bonferroni post-hoc correction (A, C) or Student’s t test (D, E). *P < 0.05, **P < 0.01, ***P < 0.001; ns, not significant.

**Fig. 3.**
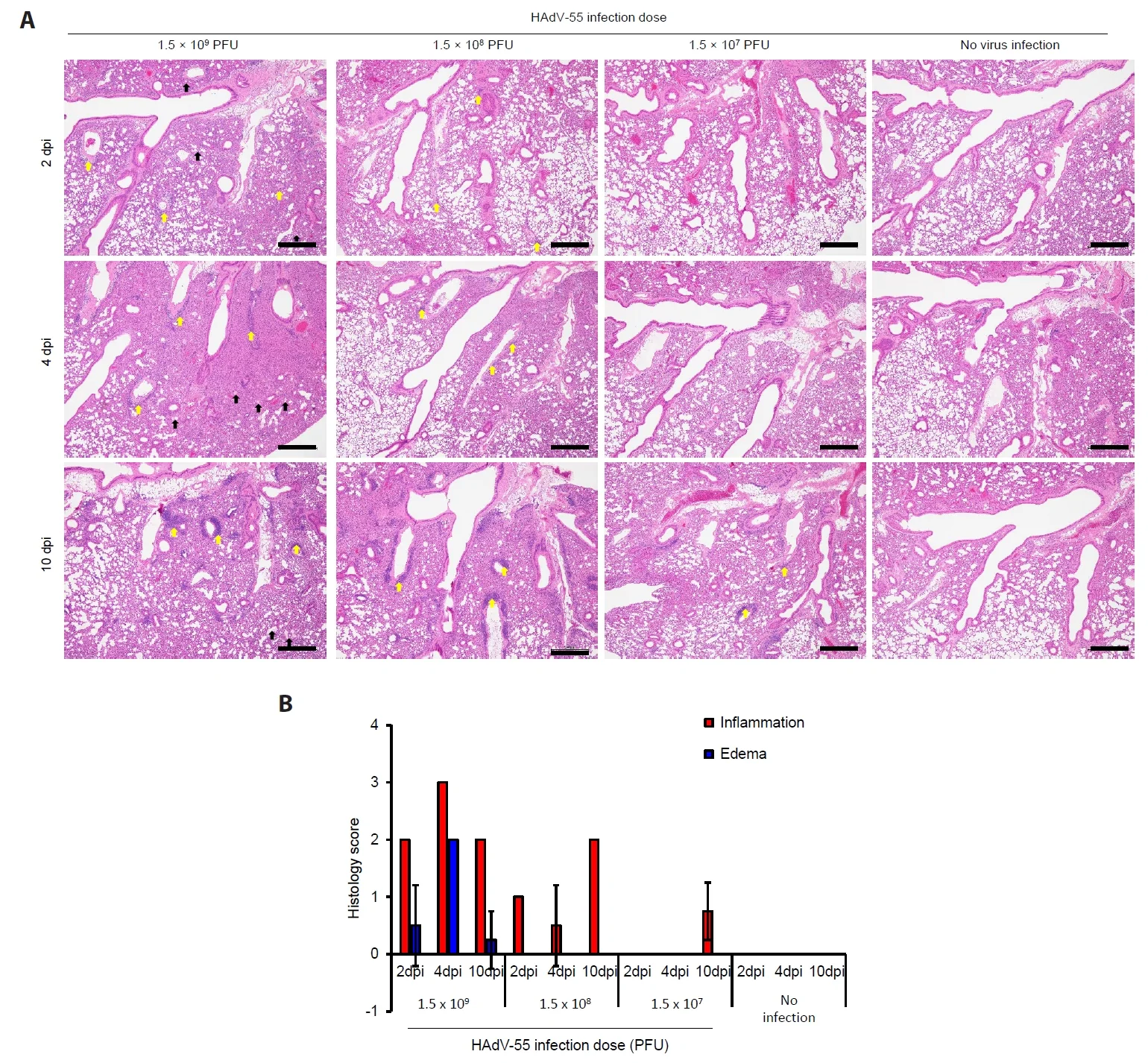
Histopathological analysis of lung tissues following HAdV-55 infection in CD46/DSG-2 transgenic mice. Lung tissues were collected from mice infected with HAdV-55 at doses of 1.5 × 10^7^, 1.5 × 10^8^, or 1.5 × 10^9^ PFU at 2, 4, and 10 days post-infection (dpi) (n = 2 mice were included at each collection time point), and processed for hematoxylin and eosin (H&E) staining. (A) Representative histological images are shown. Yellow arrows, inflammation; Black arrows, edema. (B) Histopathological scoring of inflammation (red bars) and edema (blue bars) was performed based on standardized criteria. Data are presented as Mean ± SD, demonstrating a dose-dependent increase in lung pathology following HAdV-55 infection, with peak severity observed at 2–4 dpi. Scale bars = 500 μm.

**Fig. 4.**
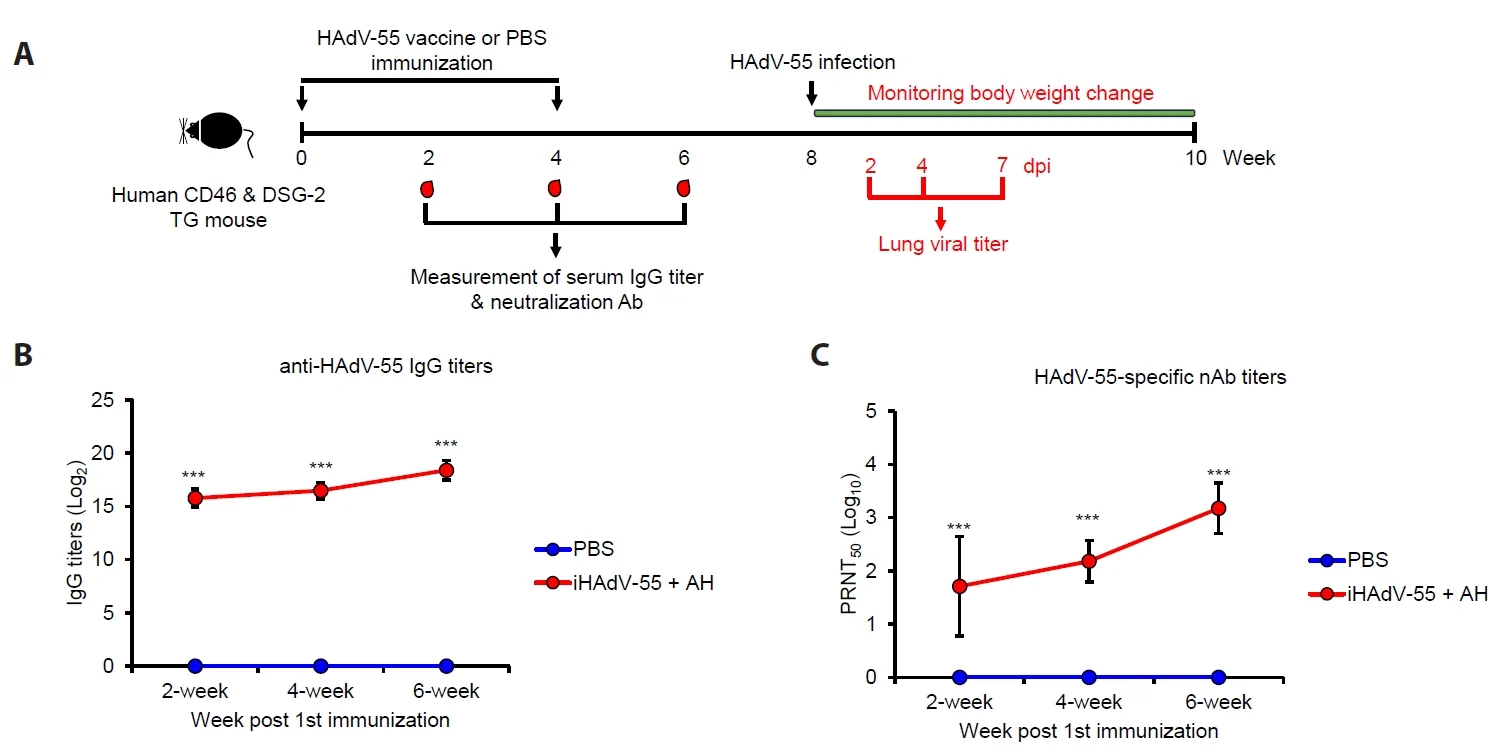
Immunization schedule and humoral immune responses induced by iHAdV-55 in CD46 and DSG-2 expressing TG mouse. (A) Human CD46/DSG-2 transgenic mice (n = 14/group) were immunized with inactivated HAdV-55 vaccine or PBS at weeks 0 and 4. Serum IgG and neutralizing antibodies were measured at weeks 2, 4, and 6. At week 8, mice were challenged with HAdV-55 and monitored for body weight changes. Lung tissues were collected at 2, 4, and 7-day post infection (dpi) for viral titer determination (n = 3 mice/time point). (B) HAdV-55-specific IgG titers measured at weeks 2, 4, and 6 post-immunizations. (C) HAdV-55-specific nAb titers (PRNT₅₀) at indicated time points. Data are presented as Mean ± SD. ***P < 0.001 compared to PBS group.

**Fig. 5.**
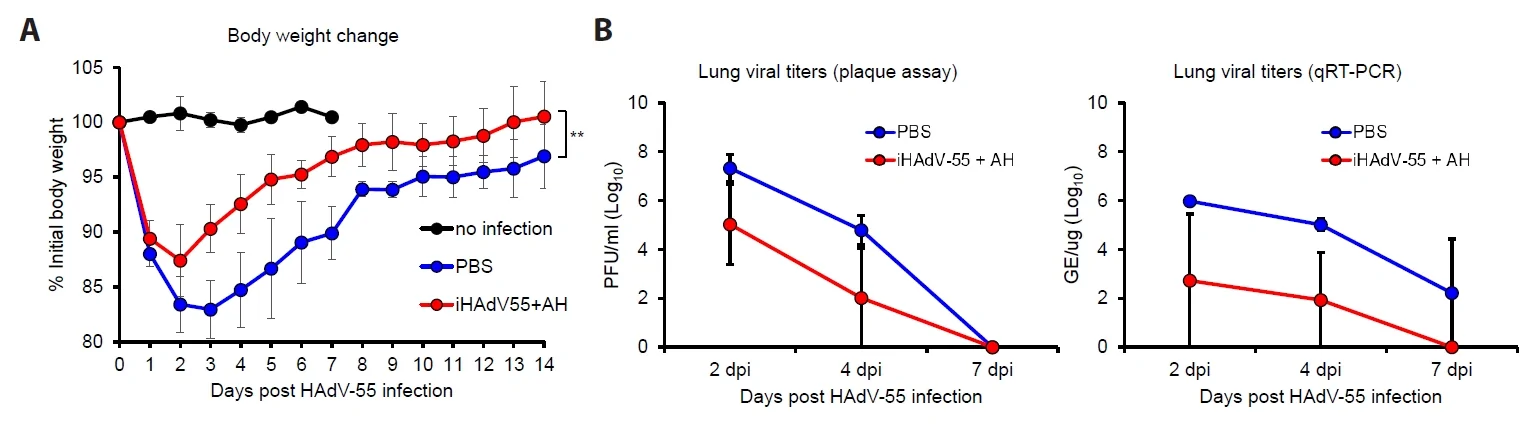
Protective efficacy of iHAdV-55 vaccination following HAdV-55 challenge. (A) Body weight changes following HAdV-55 challenge. Mice were immunized with inactivated HAdV-55 vaccine formulated with aluminum hydroxide (iHAdV-55 + AH) or PBS and challenged with HAdV-55. Body weight was monitored daily and is presented as a percentage of the initial body weight. The no-infection group served as an unchallenged control. Data are presented as Mean ± SD (n = 5 mice for the no-infection group; n = 14 mice/group before challenge). (B) Lung viral loads following HAdV-55 challenge, determined by plaque assay (left) and qRT-PCR (right). Lung tissues were collected at 2, 4, and 7 days post-infection (dpi) for viral titer determination. **P < 0.01. Data are presented as Mean ± SD (n = 3 mice/time point).

**Table 1.** Primers for genotyping of transgenic mouse

Primer name	Sequences	Product size
CMV-F	TCCTAGGCCTACTTACAAGCC	5,178 bp
BGH-R1	TCTTGGACAGATCCTCTCCCT	
hCD46-F1	GTGGATAGCGGTTTGACTCAC	450 bp
BGH-R1	TCTTGGACAGATCCTCTCCCT	
WT-F1	CACCTGCCCTGAGTGTTTCTT	325 bp
WT-R1	GATTTCCCTCTCAGCTGTGGT	

## Supplementary Information

Fig. S1. Pulmonary histopathological changes after live or inactivated HAdV-55 inoculation in human CD46/DSG-2 transgenic mice. (A) Human CD46/DSG-2 transgenic mice were intranasally inoculated with PBS, live HAdV-55, or inactivated HAdV-55 and euthanized at 2 and 4 dpi for lung histopathological analysis. (B) Representative H&E-stained lung sections are shown. Yellow arrows: inflammation, Black arrows: Edema, Quantitative histology scores for inflammation and edema are shown on the right. Scale bars = 500 μm. Data are presented as Mean ± SD.
