# Supplementary material for: Establishment of a CD46 and desmoglein-2 expressing mouse model for human adenovirus type 55 vaccine evaluation
Source: J Microbiol. 2026 Sep 18;64(9):e2605005. doi: 10.71150/jm.2605005 (PMC13688128; doi:10.71150/jm.2605005)
Supplement: Supplementary file 1 [file jm-2605005-Supplementary-Fig-S1.pdf]

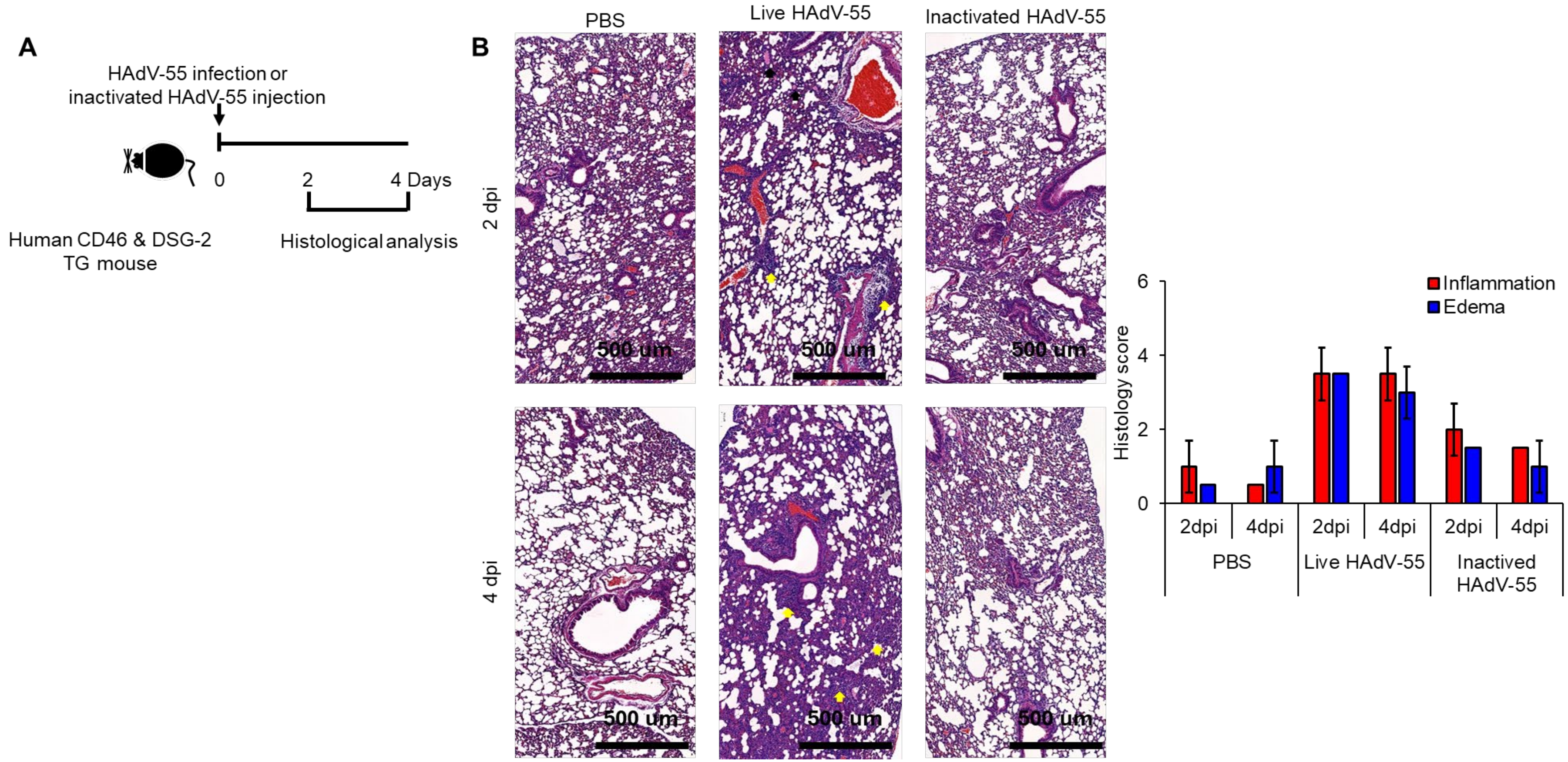

Fig. S1. Pulmonary histopathological changes after live or inactivated HAdV-55 inoculation in human CD46/DSG-2 transgenic mice. (A) Human CD46/DSG-2 transgenic mice were intranasally inoculated with PBS, live HAdV-55, or inactivated HAdV-55 and euthanized at 2 and 4 dpi for lung histopathological analysis. (B) Representative H&E-stained lung sections are shown. Yellow arrows: inflammation, Black arrows: Edema, Quantitative histology scores for inflammation and edema are shown on the right. Scale bars = 500 µm. Data are presented as Mean ± SD.
